# PrEP and the syndemic of substance use, violence, and HIV among female and male sex workers: a qualitative study in Kisumu, Kenya

**DOI:** 10.1002/jia2.25266

**Published:** 2019-04-15

**Authors:** Angela R Bazzi, Kelly Yotebieng, Sophie Otticha, Grace Rota, Kawango Agot, Spala Ohaga, Jennifer L Syvertsen

**Affiliations:** ^1^ Department of Community Health Sciences Boston University School of Public Health Boston MA USA; ^2^ Department of Anthropology Ohio State University Columbus OH USA; ^3^ Impact Research & Development Organization Kisumu Kenya; ^4^ Kenya Medical Research Institute Kisumu Kenya; ^5^ Department of Anthropology University of California Riverside CA USA

**Keywords:** HIV infections, pre‐exposure prophylaxis, alcohol drinking, sex workers, violence, Kenya

## Abstract

**Introduction:**

Female and male sex workers experience heightened vulnerability to HIV and other health harms that are compounded by substance use, physical and sexual violence, and limited access to health services. In Kisumu, Kenya, where sex work is widespread and substance use is a growing public health concern, offering pre‐exposure prophylaxis (PrEP) for HIV prevention could help curtail the HIV epidemic. Our study examines “syndemics,” or mutually reinforcing epidemics of substance use, violence and HIV, in relation to PrEP acceptability and feasibility among female and male sex workers in Kenya, one of the first African countries to approve PrEP for HIV prevention.

**Methods:**

From 2016 to 2017, sex workers in Kisumu reporting recent alcohol or drug use and experiences of violence participated in qualitative interviews on HIV risk and perspectives on health service needs, including PrEP programming. Content analysis identified themes relating to PrEP knowledge, acceptability, access challenges and delivery preferences.

**Results:**

Among 45 female and 28 male sex workers, median age was 28 and 25 respectively. All participants reported past‐month alcohol use and 91% of women and 82% of men reported past‐month drug use. Violence was pervasive, with most women and men reporting past‐year physical (96% women, 86% men) and sexual (93% women, 79% men) violence. Concerning PrEP, interviews revealed: (1) low PrEP knowledge, especially among women; (2) high PrEP acceptability and perceived need, particularly within syndemic contexts of substance use and violence; and (3) preferences for accessible, non‐stigmatizing PrEP delivery initiatives designed with input from sex workers.

**Conclusions:**

Through a syndemic lens, substance use and violence interact to increase HIV vulnerability and perceived need for PrEP among female and male sex workers in Kisumu. Although interest in PrEP was high, most sex workers in our sample, particularly women, were not benefiting from it. Syndemic substance use and violence experienced by sex workers posed important barriers to PrEP access for sex workers. Increasing PrEP access for sex workers will require addressing substance use and violence through integrated programming.

## 1 Introduction

Globally, sex workers experience increased risk of HIV, sexually transmitted infections (STI), and other adverse health outcomes, yet often have limited access to vital health services 1. Female sex workers have an estimated 13 times higher risk of HIV acquisition than the general population of women 2. Male sex workers have over 20 times higher HIV prevalence than the general male population 3 but have received much less research and programmatic attention than female sex workers 4. Most male sex workers exchange sex with other men, but HIV prevention interventions targeting “men who have sex with men” (MSM) may not reach those who do not identify as gay or as “sex workers” 5, 6. In sub‐Saharan Africa, where the majority of the HIV epidemic is concentrated, sex work among women and men is criminalized, stigmatized and often driven by poverty 7, 8, 9. Thus, female and male sex workers require HIV programming that is responsive to their complex lived experiences.

Further compounding sex workers' vulnerability to HIV infection, sexual exchange is often negotiated in alcohol‐serving establishments, where many female and male sex workers engage in alcohol and drug use that can exacerbate their risks for HIV and other health harms 10. Research from Kenya, which has an entrenched HIV epidemic, has linked substance use with unprotected sex 11 and physical and sexual violence among female sex workers 12, 13. Emerging research has also identified a high prevalence of substance use and violence victimization among male sex workers in Kenya 14, 15, as well as multiple forms of stigma that limit their use of existing HIV prevention services 16.

Recognizing the need for new strategies to fight its persistent HIV epidemic, in 2017, Kenya became one of the first African countries to make antiretroviral pre‐exposure prophylaxis (PrEP) available for HIV prevention among “key populations,” including sex workers, MSM and people who inject drugs. Although these categories could include high‐risk sex workers, to date, PrEP service delivery has yet to address the specific contexts of co‐occurring alcohol and drug use and violence victimization experienced in sex worker populations. Given that social and behavioural factors significantly influence PrEP utilization 17, 18, the success of PrEP for HIV prevention in sex worker populations affected by substance use and violence will require understanding their attitudes on, experiences with and preferences for PrEP service delivery.

This qualitative study focused on “syndemics,” or mutually reinforcing epidemics of substance use, violence, and HIV among female and male sex workers in Kisumu County, western Kenya. Syndemic theory refers to the clustering of two or more health conditions. This perspective highlights how synergistic interactions among psychosocial and biological conditions underlie patterns of disease clustering within local political, economic and social contexts, helping to identify needs for comprehensive healthcare services for those affected 19, 20.

Sex work is criminalized in Kenya, but its widespread practice in Kisumu reflects economic inequalities and limited opportunities. While HIV prevalence in Kisumu County reaches 18.7% (vs. 5.6% nationally) 21, it is disproportionately concentrated among key populations. As of 2008, the latest estimate available, there were nearly 1700 female sex workers in Kisumu, among whom HIV prevalence reached 56.5% 11. While no similar estimates are available for male sex workers, recent studies with MSM in Kisumu have documented widespread substance use, mental health conditions, and physical and sexual violence victimization that could exacerbate HIV risk 22. Drawing from syndemic theory, we explored how substance use, violence and HIV risk shape PrEP acceptability, access and intervention needs among sex workers in Kisumu.

## 2 Methods

### 2.1 Study design and population

From 2016 to 2017, to recruit sex workers, peer educators from local HIV prevention organizations conducted outreach and targeted sampling at sex work “hotspots” 23. Snowball sampling later involved asking enrolled participants to refer other sex workers to the study 24. We obtained oral consent to screen for eligibility, which included being ≥18 years old and reporting: past‐month trading of sex for money, alcohol, drugs or other material items; past‐month “problematic” alcohol use (binge drinking of ≥5 drinks or being drunk “most” or “all” of the time when drinking) or any injection or non‐injection drug use; and past‐year physical or sexual violence from intimate partners, clients or police. We used purposive sampling methods to ensure that our sample was diverse in age and substance use 25, which necessitated additional recruitment of women. Eligible participants provided written consent. Ethics review boards at The Ohio State University and Maseno University in Kisumu, Kenya, approved all study protocols, which were translated into Kiswahili and Dholuo.

### 2.2 Data collection

Trained, multilingual interviewers conducted semi‐structured interviews lasting up to 90 minutes in private offices in participants' language of choice (English, Swahili, Dholuo). Interviewers assured confidentiality and no names or identifiers were recorded. Semi‐structured interviews explored sex work, substance use, experiences of violence and perspectives on health needs (e.g. “How did you become involved in sex work? Have there been times when you have experienced violence?”). Regarding PrEP, we asked if participants had ever heard of it, and if not, provided a basic description. We then asked, “What do you think about PrEP for HIV prevention for women [men] who do sex work? Is this something you would be interested in? Why [not]?” We then explored personal interest in PrEP and preferred service delivery locations and providers. Finally, we noted that PrEP is effective and promotes adherence when taken around the same time every day and asked, “What do you think about this? How easy or difficult would it be for you to take PrEP every day?” Interviewers referred interested individuals to organizations offering PrEP for more information, and followed up as necessary. Interviews were audio recorded, transcribed verbatim and translated into English for text analysis following a structured protocol 26. We continued interviewing until we reached saturation 27, the point at which we determined as a team that we were repeatedly hearing similar information across interviews. We then decided that our sample size was sufficient to answer our main study questions (i.e. additional interviews would not yield important new insights) around health needs in syndemic contexts.

### 2.3 Data analysis

Codebook development involved several steps 28. First, the research team read through selected transcripts and independently generated lists of initial themes based on the interview guide content (e.g. PrEP knowledge) 29. The team met to discuss, operationalize and organize themes into a set of codes. One research assistant applied final codes to all transcripts using MAXQDA. The PI assessed code application for consistency and met regularly with the RA to discuss and clarify any issues. Content analysis allowed us to identify the most common experiences regarding PrEP knowledge, use, acceptability and preferences for service delivery within contexts of syndemic substance use and violence. We adopted a constant comparative method 30 to assess similarities and differences across women's and men's experiences. Findings are illustrated with quotes and pseudonyms are used to protect confidentiality.

## 3 Results

### 3.1 Sample characteristics

Among 45 women and 28 men (n = 73 sex workers), women were slightly older than men (median 28 vs. 25 years; Table 1). Attesting to the considerable mobility of both groups, most had lived outside of Kisumu (91% of women, 89% of men). All participants reported past‐month alcohol use, with 56% of women and 71% of men reporting “always” or “often” drinking to the point of becoming drunk. More women than men reported ever injecting drugs (49% vs. 36%) and recently using heroin (47% vs. 32%). Other past‐month drug use was common, as 91% of women and 82% of men used drugs, including *bhang* (marijuana; 80% women, 75% men), *miraa* (khat; 27% women, 61% men), and non‐prescribed medications (18% women, 43% men). Violence victimization was pervasive, with the majority of participants reporting past‐year physical (96% women, 86% men) and sexual (93% women, 79% men) violence. HIV prevalence was high, with 31% of both women and men self‐reporting HIV‐positive status.

**Table 1:** Characteristics of sex workers in Kisumu, Kenya (n = 73)

Variable	Women (n = 45)	Men (n = 28)
Age in years; median (interquartile range)	28 (18 to 42)	25 (19 to 41)
Born in Kisumu	21 (47%)	21 (75%)
Ever lived outside of Kisumu	41 (91%)	25 (89%)
High school education or greater^a^	14 (44%)	24 (86%)
Alcohol use, past month	45 (100%)	28 (100%)
“Always/often” drunk when using alcohol	25 (56%)	20 (71%)
Ever used drugs	42 (93%)	28 (100%)
Ever injected drugs	22 (49%)	10 (36%)
Drug use, past month (any)	41 (91%)	23 (82%)
*Bhang* (marijuana)	36 (80%)	21 (75%)
*Miraa* (khat)	12 (27%)	17 (61%)
Brown sugar and/or white crest (heroin)	21 (47%)	9 (32%)
Prescription pills (non‐medical use)	8 (18%)	12 (43%)
Experienced physical violence in past year (hitting, punching, or any other bodily harm)	43 (96%)	24 (86%)
Experienced sexual violence, past year (unwanted touching, forced sex, rape)	42 (93%)	22 (79%)
HIV‐positive (self‐report)^b^	14 (31%)	8 (31%)
Ever heard of PreP	9 (20%)	24 (86%)
Ever tried PrEP^c^	1 (3%)	7 (39%)

^a^Of the 32 women who responded to the question; ^b^two men did not disclose their HIV status; ^c^percentage calculated of the 31 HIV‐negative women and 18 HIV negative men.

Through qualitative interviews, we identified the following key themes: (1) differences between women and men in PrEP knowledge and experience; (2) high acceptability and perceived need for PrEP within syndemic contexts of sex work, substance use and violence; and (3) preferences for greater sex worker involvement in PrEP service delivery.

#### 3.1.1 PrEP knowledge and use

Stark differences emerged between women and men in PrEP knowledge and experience. Despite several major PrEP clinical trials and demonstration projects having been conducted in Kenya (including in the western region) 31, only 20% (n = 9) of women had even heard of PrEP. Many women confused PrEP with post‐exposure prophylaxis (PEP), which they were familiar with due to the frequency of rape and sexual violence and the availability of PEP in local clinics. After the interviewer explained PrEP, women expressed strong interest in trying it and asked numerous questions, including where to obtain it. Esther, 27, one of the few women who had previously heard of PrEP, described being turned away when trying to access it, illustrating the discretion of healthcare providers in determining PrEP “eligibility:”

He [healthcare worker] was asking about the high risk of being infected. I told him, if I am a sex worker, definitely I am at high risk, even though I use condoms…one day it can burst. If I get PrEP, I am safe. But because I was using condoms, I was not eligible for PrEP; that is what he told me.

In contrast, most men (86%, n = 24) knew about PrEP. Of the seven HIV‐negative men who had experience using PrEP, four were using it at the time of the interview; all four held favourable views of it. While describing PrEP as “awesome,” two mentioned having to overcome unpleasant side effects:

[Taking PrEP] is hard but it has benefits and challenges for a few weeks because…. It's like you are tired and drowsy and you have a bloated stomach, but [the side effects] end.

Side effects caused two men in our sample to discontinue their PrEP use. Two HIV‐positive men were involved with local organizations as “PrEP ambassadors,” helping educate other men about PrEP and “mobilize” them into services. They also reported that side effects caused several of their peers to stop using PrEP. The other individual in our study who discontinued PrEP feared that because he received PrEP at a clinic that also provided HIV care, people would assume him to be HIV‐positive and taking ARVs.

#### 3.1.2 Acceptability and perceived need for PrEP in syndemic contexts of sex work

Both women and men described the multifaceted social, legal and health hazards of sex work. Overall, narratives revealed high acceptability of and perceived need for PrEP due to syndemic conditions wherein substance use and violence exacerbated HIV risk. Given the high HIV prevalence in Kisumu, most participants expressed concern at not knowing clients' HIV status. Furthermore, many participants had clients who refused to use condoms despite not disclosing their status.

The pervasiveness of heavy alcohol use within sex work contexts further increased sex workers' perceived need for PrEP. Women and men described frequently drinking alcohol with clients in bars and clubs prior to having sex, which sometimes led them to “black out” and “forget” about condoms or agree to have condomless sex for higher pay. Combined with their heavy drinking, poverty and the need to support children rendered women particularly vulnerable to having condomless sex, as described by Lena, age 26:

I think [sex workers] should use [PrEP] because this is the work that they do. Since you really want money, you might end up having sex with a client without protection. [Or] you can be in a situation whereby the client plans to misuse you or try to take advantage of you after taking alcohol. You need to have [PrEP].

In such cases in which sex workers became “so drunk they lose their senses,” PrEP would provide an extra level of protection from HIV.

Moreover, women discussed how the likelihood of sexual violence increased in contexts of sex work and heavy drinking, including forced condomless sex:

It [sex work] is a job that can cause your death. Someone can stab me with a knife the moment I leave here. Or someone can have sex with me without a condom, or someone can rape me. They can come as a group of three people, break the door and all the three people can rape me.—Sera, age 21

Our risks come in that the condom might burst. Maybe I can be raped somewhere by someone whose status I don't know. It [PrEP] will therefore protect us.—Rose, age 27

Although sexual violence was described more frequently by women, men also viewed PrEP as a valuable prevention tool in contexts of alcohol use and violence victimization:

[PrEP] is good [because] there are some clients who can rape you, and alcohol is stronger than some sex workers. After he has taken so much alcohol and he is drunk, when he goes to the room he will not remember to use condom[s], so I think it is good for sex workers.—Steven, age 23

Overall, perceived acceptability of and need for PrEP was high among female and male sex workers.

#### 3.1.3 Preferences for PrEP delivery

Given the syndemic health risks experienced by sex workers in Kisumu, maximizing the HIV prevention potential of PrEP will require understanding their preferences for PrEP delivery. Across interviews, participants stressed the importance of accessing PrEP from non‐judgmental providers within service delivery settings that were “friendly” to sex workers. Participants did not want to be discriminated against due to their sexual orientation, substance use, or engagement in sex work. While some women described feeling comfortable interacting with non‐judgmental clinical providers in health centres, others explained that fellow sex workers would be more appropriate sources of PrEP information, referrals and services because they would understand the multifaceted risks of sex work and could converse comfortably about PrEP in the context of those risks:

It should be a fellow sex worker. We can tell her everything that we are going through. If you look at us, not all of us are fools. There are those who have reached the university level. They can be trained a little and later come to do such work. We want our freedom. What I mean by freedom is a place where I will enter and talk to them the way I want.—Rose, age 27

Given their understanding of the contextual realities of sex work, fellow sex workers could also help women address potential challenges with PrEP adherence:

If you want to help people then it is good that you [employ] people who are like [us] because they have been in the field [practicing sex work] and they understand our lives. So it is good that you employ those who have done the same job [sex work] to dispense the drug for them … And even the counselors should also be sex workers.—Nancy, age 31

As noted above, some men in our sample already had experience discussing PrEP with their peers as “PrEP ambassadors.” These men stressed the importance of PrEP provision involving healthcare workers who were comfortable serving males who had male sex partners. When asked about who should deliver PrEP, Johnson, age 32, responded:

Make sure they are people who are not homophobic, that they are people who are friendly to gay people. Because if they are homophobic, they are going to make remarks that will make people be angry. And then people will not be accessing that kind of service from that place.

Some men also specified that providers should be comfortable discussing same‐sex sexual behaviours and sex work in non‐judgmental ways. As Samuel, age 26, said, providers unfamiliar with same‐sex behaviours should not provide PrEP to male sex workers because they would “not know anything about sex work.”

Importantly, women and men emphasized that PrEP providers should understand sex workers' specific syndemic vulnerabilities as described above. Participants provided specific suggestions for improving PrEP uptake among sex workers. First, due to the concentration of sex work and drinking establishments catering to sex work, PrEP should be provided through convenient, confidential spaces in these areas. Second, PrEP services should be sufficiently flexible and involve creative strategies to accommodate the high mobility of many sex workers (e.g. mobile PrEP services). Finally, due to the myriad health concerns of female and male sex workers with substance use and violence victimization, efforts to deliver PrEP should be holistic and integrated with other health services. According to Jackson, age 30:

We need facilities that work 24 hours a day. Even if you are raped, you can easily rush to the facility. I also said that we needed people to fight for our rights…Economic empowerment, security training, HIV prevention sensitization, training on behaviour change and risk reduction. [Sex workers] also need training on drugs and substance abuse. You need to create employment.

## 4 Discussion

In the context of an ongoing HIV epidemic in Kenya, national guidelines recommending PrEP for HIV prevention among key populations carry enormous potential to curb the epidemic. In Kenya, where multiple PrEP projects are ongoing, an estimated 53,000 to 54,000 individuals are currently prescribed PrEP 32. In regions shouldering the burden of the HIV epidemic like Kisumu, understanding how syndemic conditions of substance use and violence increase sex workers' vulnerability to HIV and ability to adopt new prevention technologies like PrEP will be critical 33. Viewing this situation through a syndemic lens, which conceptualizes how multiple health conditions synergistically reinforce each other 21, 22, can help address sex workers' health and social needs within local contexts 34. We provide several recommendations for improving the impact of PrEP for HIV prevention among women and men engaged in sex work.

First, we found differences in PrEP knowledge and use between women and men. While men had specific concerns about side effects and being viewed as HIV‐positive if taking PrEP, women lacked even basic access to PrEP information and services. This is particularly concerning because young Kenyan women have the highest HIV incidence nationally 19, and sex work and syndemic vulnerabilities stemming from substance use and violence further increase HIV risk. Since collecting our data, a local sex worker organization has responded to women's exclusion by conducting PrEP outreach with sex workers and launching radio media campaigns to increase PrEP awareness and reduce stigma around its use (unpublished fieldnotes). In coordination with ongoing efforts by the Kenya Ministry of Health, additional organizations in Kisumu now offer PrEP to expand access to multiple populations, including additional safe spaces for MSM and men who engage in sex work. As PrEP continues to be scaled up across Kenya, continued involvement of sex workers in raising awareness and reducing stigma will be critical to its success.

Second, we found high acceptability and perceived need for PrEP within contexts of sex work, substance use and violence. Approaching questions of PrEP access and delivery from this syndemic perspective helps emphasize the interconnectedness of HIV and other health and social concerns and highlights the need for more comprehensive and integrated services for sex workers. As our study found, entrenched substance use—particularly heavy drinking—and co‐occurring physical and sexual violence and underlying poverty all interact to increase HIV risk and PrEP need 35. Furthermore, increasingly prevalent drug use in this region of Kenya may compound the social stigma already surrounding sex work, resulting in further marginalization of sex workers 36. We recommend that facilities offering PrEP link with the growing number of psychosocial health services in Kisumu to address these complex issues, including organizations that focus on gender‐based violence, addiction and mental health issues (unpublished fieldnotes). A community‐based “technical working group” that joins all parties together, including sex workers, could collaboratively brainstorm around PrEP challenges and share resources, as this model has been helpful in coordinating efforts towards other dimensions of the local HIV epidemic. Our findings reflect the need for biomedical prevention tools to be delivered with consideration of sex workers' “real world” vulnerabilities 37.

Finally, as found elsewhere 38, sex workers in our study emphasized the need for non‐judgmental PrEP service delivery. While women and men worried about stigma against sex work, men were particularly concerned about homophobia and provider discomfort with same‐sex behaviours. We suggest that stigma is another important factor in creating syndemic vulnerability, which has also been identified as major barrier to PrEP uptake across marginalized populations 39. To ensure community ownership of new HIV prevention technologies, sex workers experiencing syndemic substance use and violence should be involved in PrEP intervention development and implementation 40. Innovative PrEP intervention strategies could include training peer sex workers in PrEP outreach (e.g. “PrEP ambassadors”). Well‐trained sex workers who are actively taking PrEP and aware of the syndemic risks experienced by fellow sex workers could effectively speak to overcoming challenges, including side effects, alcohol use and concerns about stigma, as well as provide peer‐based adherence support. Importantly, all of the above efforts would benefit from broader efforts to change the structural conditions that criminalize sex work and render substance use and violence so pervasive in the first place 1.

Our study has limitations. Our sample was purposively selected from a high‐risk population and may over‐represent levels of drug and alcohol use among sex workers. We also collected data during a time of rapid changes in PrEP policy, and our results reflect only the very early stages of dissemination. We provided very general information about PrEP; future, in‐depth research is needed to explore attitudes regarding initial clinical screening, side effects, ongoing STI screening, sexual and reproductive health needs, and potential social implications of using PrEP. Nevertheless, our study provides rare insight into the experiences of female and male sex workers who report substance use and violence who to date have largely been excluded from PrEP research and programmes.

## 5 Conclusions

Given the interlinked nature of substance use, violence and HIV vulnerability among sex workers in Kenya, our findings suggest that successful implementation of HIV prevention technologies for vulnerable populations can be strengthened using a syndemic perspective 18, 38, 41. PrEP research and programming efforts should engage sex workers who use alcohol and drugs who have not yet been included in research or outreach efforts 33. As Kenya continues to scale up PrEP delivery as part of comprehensive national HIV programming, soliciting the perspectives of key populations experiencing syndemic HIV vulnerability will be critical.

## Competing interests

The authors declare no conflict of interest.

## Authors' contributions

JLS designed the research and collected data. SO and GR collected data. KAY, JLS and ARB analysed the data. KAY, JLS and ARB wrote the first draft of the paper. All authors contributed to and approved the final manuscript.

## References

[1] Shannon K , Strathdee SA , Goldenberg SM , Duff P , Mwangi P , Rusakova M , et al. Global epidemiology of HIV among female sex workers: influence of structural determinants. Lancet. 2015;385(9962):55–71. 25059947 10.1016/S0140-6736(14)60931-4 PMC4297548

[2] Baral SD , Beyrer C , Muessig K , Poteat T , Wirtz AL , Decker MR . Burden of HIV among female sex workers in low‐income and middle‐income countries: a systematic review and meta‐analysis. Lancet Infect Dis. 2012;12:538–49. 22424777 10.1016/S1473-3099(12)70066-X

[3] Oldenburg CE , Perez‐Brumer AG , Reisner SL , Mattie J , Bärnighausen T , Mayer KH , et al. Global burden of HIV among men who engage in transactional sex: a systematic review and meta‐analysis. PLoS One. 2014;9(7):e103549. 25068720 10.1371/journal.pone.0103549 PMC4113434

[4] Baral SD , Friedman MR , Geibel S , Rebe K , Bozhinov B , Diouf D , et al. Male sex workers: practices, contexts, and vulnerabilities for HIV acquisition and transmission. Lancet. 2015;385(9964):260–73. 25059939 10.1016/S0140-6736(14)60801-1 PMC4504188

[5] Parker R , Aggleton P , Perez‐Brumer AG . The trouble with ‘Categories': rethinking men who have sex with men, transgender and their equivalents in HIV prevention and health promotion. Glob Public Health. 2016;11(7–8):819–23. 27193965 10.1080/17441692.2016.1185138

[6] Kaplan RL , Sevelius J , Ribeiro K . In the name of brevity: the problem with binary HIV risk categories. Glob Public Health. 2016;11(7–8):824–34. 26824592 10.1080/17441692.2015.1136346 PMC5140888

[7] Ngugi E , Roth E , Mastin T , Nderitu MG , Yasmin S . Female sex workers in Africa: epidemiology overview, data gaps, ways forward. SAHARA J. 2012;9:148–53. 23237069 10.1080/17290376.2012.743825 PMC4560463

[8] Scheibe A , Drame FM , Shannon K . HIV prevention among female sex workers in Africa. SAHARA J. 2012;3:167–72. 10.1080/17290376.2012.743809 23237073

[9] Scorgie F , Vasey K , Harper E , Richter M , Nare P , Maseko S . Human rights abuses and collective resilience among sex workers in four African countries: a qualitative study. Global Health. 2013;9(1):33. 23889941 10.1186/1744-8603-9-33 PMC3750273

[10] Li Q , Li X , Stanton B . Alcohol use among female sex workers and male clients: an integrative review of global literature. Alcohol Alcohol. 2010;45(2):188–99. 20089544 10.1093/alcalc/agp095 PMC2842106

[11] Vandenhoudt HM , Langat L , Menten J , Odongo F , Oswago S , Luttah G , et al. Prevalence of HIV and other sexually transmitted infections among female sex workers in Kisumu, western Kenya, 1997 and 2008. PLoS One. 2013;8(1):e54953. 23372801 10.1371/journal.pone.0054953 PMC3553007

[12] Chersich M , Luchters S , Malonza I , Mwarogo P , King'Ola N , Temmerman M . Heavy episodic drinking among Kenyan female sex workers is associated with unsafe sex, sexual violence and sexually transmitted infections. Int J STD AIDS. 2007;18(11):764–9. 18005511 10.1258/095646207782212342

[13] Pack AP , L'Engle K , Mwarogo P , Kingola N . Intimate partner violence against female sex workers in Mombasa, Kenya. Cult Health Sex. 2014;16(3):217–30. 10.1080/13691058.2013.857046 24329103

[14] Micheni M , Rogers S , Wahome E , Darwinkel M , Van Der Elst E , Gichuru E , et al. Risk of sexual, physical and verbal assaults on men who have sex with men and female sex workers in coastal Kenya. AIDS. 2015;29 Suppl 3:S231. 26562812 10.1097/QAD.0000000000000912 PMC4706373

[15] Muraguri N , Tun W , Okal J , Broz D , Raymond HF , Kellogg T , et al. HIV and STI prevalence and risk factors among male sex workers and other men who have sex with men in Nairobi, Kenya. J Acquir Immune Defic Syndr. 2015;68(1):91–6. 25501346 10.1097/QAI.0000000000000368 PMC4973514

[16] Anderson AM , Ross MW , Nyoni JE , McCurdy SA . High prevalence of stigma‐related abuse among a sample of men who have sex with men in Tanzania: implications for HIV prevention. AIDS Care. 2015;27(1):63–70. 25162483 10.1080/09540121.2014.951597 PMC5067008

[17] Eakle R , Bourne A , Jarrett C , Stadler J , Larson H . Motivations and barriers to uptake and use of female‐initiated, biomedical HIV prevention products in sub‐Saharan Africa: an adapted meta‐ethnography. BMC Public Health. 2017;17(1):968. 29258455 10.1186/s12889-017-4959-3 PMC5738143

[18] Van der Elst EM , Mbogua J , Operario D , Mutua G , Kuo C , Mugo P , et al. High acceptability of HIV pre‐exposure prophylaxis but challenges in adherence and use: qualitative insights from a phase I trial of intermittent and daily PrEP in at‐risk populations in Kenya. AIDS Behav. 2013;17(6):2162–72. 23080358 10.1007/s10461-012-0317-8 PMC3690654

[19] Singer M , Clair S . Syndemics and public health: reconceptualizing disease in bio‐social context. Med Anthropol Q. 2003;17:423–41. 14716917 10.1525/maq.2003.17.4.423

[20] Singer M . Introduction to syndemics: a critical systems approach to public and community health. San Fransisco, CA: Jossey‐Bass; 2009.

[21] NASCOP . Kenya AIDS Response Progress Report 2014: Progress towards Zero. Nairobi: National AIDS Control Council; 2014.

[22] Kunzweiler CP , Bailey RC , Okall DO , Graham SM , Mehta SD , Otieno FO . Depressive symptoms, alcohol and drug use, and physical and sexual abuse among men who have sex with men in Kisumu, Kenya: the Anza Mapema Study. AIDS Behav. 2017;22(5):1517–1529. 10.1007/s10461-017-1941-0 29079946

[23] Watters J , Biernacki P . Targeted sampling: options for the study of hidden populations. Soc Probl. 1989;36(4):416–30.

[24] Schensul JJ , LeCompte MD , Trotter RT II , Cromley EK , Singer M . Mapping Social Networks, Spatial Data, and Hidden Populations. Walnut Creek: AltaMira Press; 1999.

[25] Bernard HR . Research methods in anthropology: qualitative and quantitative approaches. Walnut Creek, CA: AltaMira; 2002.

[26] McLellan E , MacQueen KM , Neidig JL . Beyond the qualitative interview: data preparation and transcription. Field Methods. 2003;15(1):63–84.

[27] Guest G , Bunce A , Johnson L . How many interviews are enough? Field Methods. 2006;18(1):59–82.

[28] MacQueen KM , McLellan E , Kay K , Milstein B . Codebook development for team‐based qualitative analysis. CAM. 1998;10(2):31–6.

[29] Ryan GW , Bernard HR . Techniques to identify themes. Field Methods. 2003;15(1):85–109.

[30] Glaser BG . The constant comparative method of qualitative analysis. Soc Probl. 1965;12(4):436–45.

[31] Murnane PM , Celum C , Nelly M , Campbell JD , Donnell D , Bukusi E , et al. Efficacy of pre‐exposure prophylaxis for HIV‐1 prevention among high risk heterosexuals: subgroup analyses from the Partners PrEP Study. AIDS. 2013;27(13):2155–60. 24384592 10.1097/QAD.0b013e3283629037 PMC3882910

[32] AVAC . PrEP Watch: Kenya. 2018. [cited 2019 Jan 27]. Available from: https://www.prepwatch.org

[33] Shannon K , Crago A‐L , Baral SD , Bekker L‐G , Kerrigan D , Decker MR , et al. The global response and unmet actions for HIV and sex workers. Lancet. 2018;392:698–710. 30037733 10.1016/S0140-6736(18)31439-9 PMC6384122

[34] Gilbert L , Raj A , Hien D , Stockman J , Terlikbayeva A , Wyatt G . Targeting the SAVA (Substance Abuse, Violence, and AIDS) syndemic among women and girls: a global review of epidemiology and integrated interventions. J Acquir Immune Defic Syndr. 2015;69 Suppl 2:S118–27. 25978478 10.1097/QAI.0000000000000626 PMC4751344

[35] Mizuno Y , Purcell DW , Knowlton AR , Wilkinson JD , Gourevitch MN , Knight KR . Syndemic vulnerability, sexual and injection risk behaviors, and HIV continuum of care outcomes in HIV‐positive injection drug users. AIDS Behav. 2015;19(4):684–93. 25249392 10.1007/s10461-014-0890-0 PMC4636202

[36] Singer M . Development, coinfection, and the syndemics of pregnancy in Sub‐Saharan Africa. Infect Dis Poverty. 2013;2(1):26. 24237997 10.1186/2049-9957-2-26 PMC4177213

[37] Syvertsen JL , Bazzi AMR , Scheibe A , Adebajo S , Strathdee SA , Wechsberg WM . The promise and peril of pre‐exposure prophylaxis (prep): using social science to inform prep interventions among female sex workers. African J Reprod Health. 2014;18(3):74–83. PMC4605136 26050379

[38] Restar AJ , Tocco JU , Mantell JE , Lafort Y , Gichangi P , Masvawure TB , et al. Perspectives on HIV pre‐and post‐exposure prophylaxes (PrEP and PEP) among female and male sex workers in Mombasa, Kenya: implications for integrating biomedical prevention into sexual health services. AIDS Educ Prev. 2017;29(2):141–53. 28467163 10.1521/aeap.2017.29.2.141 PMC5706461

[39] Golub SA . PrEP Stigma: implicit and explicit drivers of disparity. Curr HIV/AIDS Rep. 2018;15:1–8. 29460223 10.1007/s11904-018-0385-0 PMC5884731

[40] Eakle R , Bourne A , Mbogua J , Mutanha N , Rees H . Exploring acceptability of oral PrEP prior to implementation among female sex workers in South Africa. J Int AIDS Soc. 2018;21(2):e25081. 10.1002/jia2.25081 PMC5817972 29457868

[41] van der Straten A , Stadler J , Montgomery E , Hartmann M , Magazi B , Mathebula F , et al. Women's experiences with oral and vaginal pre‐exposure prophylaxis: the VOICE‐C qualitative study in Johannesburg, South Africa. PLoS One. 2014;9(2):e89118. 24586534 10.1371/journal.pone.0089118 PMC3931679

